# Non-Kolmogorovian Probabilities and Quantum Technologies

**DOI:** 10.3390/e24111666

**Published:** 2022-11-15

**Authors:** Federico Hernán Holik

**Affiliations:** Instituto de Física La Plata, CONICET-UNLP, Diagonal 113 e/63 y 64, La Plata 1900, Argentina; holik@fisica.unlp.edu.ar

**Keywords:** non-Kolmogorovian probability, quantum technologies, philosophy of quantum physics

## Abstract

In this work, we focus on the philosophical aspects and technical challenges that underlie the axiomatization of the non-Kolmogorovian probability framework, in connection with the problem of quantum contextuality. This fundamental feature of quantum theory has received a lot of attention recently, given that it might be connected to the speed-up of quantum computers—a phenomenon that is not fully understood. Although this problem has been extensively studied in the physics community, there are still many philosophical questions that should be properly formulated. We analyzed different problems from a conceptual standpoint using the non-Kolmogorovian probability approach as a technical tool.

## 1. Introduction

The development of quantum technologies is one of the greatest challenges of our time [1]. We are facing important changes that might have deep social implications. There have been incredible advances in coherently manipulating quantum systems [2,3]. Public and private investments have fueled the development of these technologies to a higher rate. All these efforts have resulted in the creation of many companies that rendered quantum devices commercially available [4]. In particular, there have been developments of quantum computers that perform tasks that are very hard for their classical counterparts [5,6,7,8,9]. The aim of this work is to highlight some questions related to the development of quantum technologies in connection to the peculiar nature of quantum probabilities that are considered relevant for the philosophy of physics.

One of the main questions that we will address is: what is the feature that makes quantum computers—and more generally, quantum technologies—so special? As we will argue (and others have stressed), the answer to this question poses deep questions about the foundations of quantum theory. We focus on the interpretation of quantum probabilities as a non-Kolmogorovian calculus. Related to this approach, the notion of quantum contextuality will play a major role.

First, we will revisit the notion of quantum randomness, which is unavoidably present in all quantum phenomena. We will argue that it is possible to understand the main quantum features as expressions of the existence of systems that instantiate a genuine non-classical probability calculus. Quantum simulators (i.e., classical systems mimicking quantum devices) lack the capability of generating genuine (quantum) contextuality. For that reason, as the simulated qubit numbers grow, they consume exponential resources that can be quantified (see for example [10]). Related to that, quantum simulators cannot be considered sources of genuine randomness.

We will describe quantum information theory as the information theory that arises when the probabilities involved are non-Kolmogorovian [11]. Quantum systems can be described as collections of classical probability distributions, whose associated Boolean algebras are intertwined in an intricate way. As a result, there is no consistent way of constructing a global classical probability distribution. In particular, we show how the algorithms run on a circuit quantum computer can be described in terms of a probabilistic computing model that relies on non-Kolmogorovian probabilities [12].

From the point of view adopted in this work, the efficiency of a quantum device can be understood as its capability of navigating the whole quantum state space. In other words: how capable it is of creating a genuine non-Kolmogorovian probability. Of course, any real device will contain imperfections, and the effect of the environment cannot be neglected, rendering quantum error correction protocols unavoidable. Therefore, the capability of realizing the whole quantum probability space in a controlled device can be attained only to a certain degree. However, this can be quantified, and recent experiments can be understood as expressions of that increased power. Here, we will argue that the ever-growing challenges that these tasks represent for classical computers can be understood as expressions of the hypotheses that classical simulators cannot be sources of genuine non-classical probabilities.

This is mainly a review work, but it also contains the proposal for interpreting the salient features of quantum technologies as consequences of non-classical quantum probabilities. Notice that this point of view is conceptually different from other approaches that attribute the reasons for quantum advantages to non-locality, or even to the many worlds. As such, our point of view is closer to those approaches that single out contextuality as the more relevant feature of quantum mechanics. Moreover, while some sections will be rather technical, others will avoid mathematical sophistication and will be written using more intuitive jargon. The chosen style of writing is related to the goal of alerting philosophers of physics about the conceptual problems that the development of quantum technologies entails, and the role played by quantum probability theory. The author hopes that this work serves as an introduction to these problems, and as motivation for further work as well. Some works related to the philosophical aspects of quantum information theory and technologies can be found in [13,14,15,16,17]. In this work, we will focus on quantum probability theory (but the reader can refer to those references for a more general discussion on the subject).

One of the main messages of this work is that we do not fully understand randomness in general, and quantum randomness in particular. By learning how to manipulate quantum probabilities, we are only looking at the tip of the iceberg of something bigger. In light of this observation, the interpretation of probabilities in terms of ignorance (and subjective probabilities in general), is a not-so-appealing point of view, since it distracts us from what seems to be the most important feature of quantum physics: the existence of irreducible randomness in nature, which is governed by mathematical laws whose meanings and properties are not yet fully understood. This problem calls for deeper efforts from the philosophy of physics community, specifically, to understand quantum contextuality and the peculiar features of quantum probability. The development of quantum technologies can provide great insight into these questions. To a great degree, the so-called quantum advantage is related to the capability of a device to explore (as much as possible) the quantum state space in a controlled way. As we argue below, this task is clearly connected to that of realizing a quantum probability model at its full strength.

The work is organized as follows. In Section 2, we start with the main philosophical assumptions behind what is perhaps the most obvious technological application of quantum theory: the development of quantum random number generators. There, we argue that empirical evidence gives support to the assumption that quantum probabilities have an irreducible ontological nature. Next, in Section 3, we fully enter the quantum formalism and revisit the formulation of quantum probability theory as a non-Kolmogorovian calculus. We also highlight the quantum features that give place to the notion of quantum contextuality. In Section 4, we discuss quantum information theory and, in particular, quantum computing, from the point of view of the non-Kolmogorovian approach. There, we argue that quantum computing is a non-Kolmogorovian version of classical probabilistic computing. We revisit some questions regarding quantum advantage and delineate some ideas that, we believe, could be interesting to develop in further works. Finally, in Section 6, we list some conclusions.

## 2. The Ideas behind Quantum Probability

There is a fact that has somewhat escaped the current philosophical debates about quantum theory: quantum physicists and engineers develop and sell quantum random number generators (QRGs) [18,19,20]. The generation of genuine randomness is crucial in many areas, such as cybersecurity, but also scientific mathematical modeling and gambling. Therefore, many efforts are invested in the development (and certification) of such devices, and several companies have them commercially available (by the time of writing this work) [21]. As usual, noise and imperfections conspire against their effectiveness. The development of QRGs is one of the most important technological applications of quantum theory and constitutes a very active field of research nowadays. It also contributes to the goal of gaining knowledge about the most fundamental aspects of nature [22,23].

What is the main working hypothesis behind the idea of devising a QRG? It is very simple: quantum systems are considered to be genuine sources of true randomness. According to this hypothesis, there will never be a way to find out the law that produces a string of random numbers generated by a quantum device (so a hacker can gain advantages during an attack) since this law simply does not exist. Where does this idea come from?

### 2.1. In the Beginning, There Was Ontological Probabilities

In his Nobel lecture [24], M. Born clearly states:

“I should like only to say this: the determinism of classical physics turns out to be an illusion, created by overrating logical-mathematical concepts. It is an idol, not an ideal in scientific research and cannot, therefore, be used as an objection to the essentially indeterministic statistical interpretation of quantum mechanics.”

A clever ’move’ of the founders of quantum theory was to realize that there was something essentially new regarding the probabilistic behavior of quantum systems. Instead of continuing to look for hidden variables and mechanisms that were ultimately responsible for the experimental outcomes, they realized that the focus should be placed on the probabilities instead. W. Heisenberg clearly realized this too, and stated [25]:

“It is no longer the objective events but rather the probabilities for the occurrence of certain events that can be stated in mathematical formulae. It is no longer the actual happening itself but rather the possibility of its happening—the potential, to employ a concept from Aristotle’s philosophy—that is subject to strict natural laws.”

However, what was the nature of the probabilistic behavior? The turn to a probabilistic description was accompanied by the intuition that there was something entirely new lurking from the depths. In the words of W. Pauli:

“It was quantum mechanics that first assumed the existence of primary probabilities in the laws of nature, which could not be reduced, by means of auxiliary hypotheses, to deterministic laws, as is possible, for instance, with the thermodynamical probabilities of classical physics. This revolutionary development is considered as final by the large majority of modern physicists, first of all by Born, Heisenberg, and Bohr, with whom I myself agree.”

([26], page 46)

He continues with a stronger assertion:

“The state of a system (object) being given, only statistical predictions can in general be made about the results of future observations (primary probability), *whereas the result of the single observation is not determined by laws, being thus an ultimate fact without cause*. This is necessary in order that quantum mechanics may be regarded as the rational generalization of classical physics, and complementarity as the generalization of causality in the narrower sense.”

([26], page 46, our emphasis)

The existence of “primary probabilities” in nature is one of the main reasons that motivate the quest for genuine randomness in technological applications. These primary probabilities would not admit a reduction to ignorance, and they would not be determined by a hidden law—one that a hacker could eventually discover. As Pauli remarks, they represent real chances that exist in nature (and not in our minds). Thus, there is no hidden mechanism to discover. Quantum experiments were assumed to involve alternatives that have results “without cause”.

The assumption that there is intrinsic randomness in nature leads to the notion of what we call *ontological probabilities*. In many applications, probabilities can be understood as the mere ignorance of an observer. As an example, one might not know the fine details of a coin toss, this being the cause of the adoption of a probabilistic description. We refer to this point of view as the *ignorance interpretation of probabilities*. The ignorance interpretation can also be accompanied by extreme skepticism. One extreme subjectivist could argue: “We don’t know. Moreover, it is meaningless to try to go beyond the mental activity of the subject. Therefore, we use a probabilistic description”. This point of view lies at the basis of many variants of the Bayesian approach to probability theory. In contrast, the ontological probabilities assumed for quantum systems have an opposite meaning. It is assumed that there is intrinsic randomness in the outside world and that we cannot predict the results of future experiments, not because we do not have access (or because we are stubbornly skeptic), but because, in reality, there is nothing to know. It is a metaphysical assumption about the ultimate nature of reality. We analyze (one more time) Heisenberg’s words [27]:

“In throwing dice we do not know the fine details of the motion of our hands which determine the fall of the dice and therefore we say that the probability for throwing a special number is just one in six. The probability wave of Bohr, Kramers, Slater, however, meant more than that; it meant a tendency for something. It was a quantitative version of the old concept of “potentia” in Aristotelian philosophy. It introduced something standing in the middle between the idea of an event and the actual event, a strange kind of physical reality just in the middle between possibility and reality.”

How can we describe ontological probabilities in a rigorous philosophical way? There is no need to go too far. As Heisenberg intuited, the Aristotelian notion of "potentia" fits very well with what we observe in the quantum realm. Several authors have elaborated on that direction (for example, see [28,29,30,31,32]). A related notion is that of propensity, as in Popper’s proposal [33]. We notice that "propensitons"—understood as entities that are made of propensities—were proposed during the 1980s [34]. In this context, it is also relevant to mention the ontologies based on bundles of possible properties [35,36]. All of these approaches have the common idea in mind that, in some way or another, the notion of possibility is part of a reality that is objective and external to the subject (and that it obeys laws that can be known). Therefore, the possibility ends up having an ontological status. These observations should suffice to understand the above quotations and why the belief about the existence of genuine sources of true randomness is so strong among physicists and quantum engineers.

### 2.2. Experimental Evidence Supports the Ontological Probabilities Assumption

At this point, it is important to ask: What does empirical evidence say? Most experiments involve some degree of randomness. This is ingrained in the errors that appear in the communications of experimental results. In fact, from all possible natural phenomena, it is only in a very tiny fraction that we can obtain full predictability. Of course, in classical physics, if we refine the experimental conditions, we can make the probabilistic behavior arbitrarily small. It will rarely be exactly zero but, at least, evidence suggests that in many of those systems that we call classical, finer experimental conditions will be reflected in higher predictability (at least, when we analyze them in a controlled environment). Contrarily, the unpredictability associated with quantum phenomena—after almost one hundred years of efforts—seems to be irreducible.

Unpredictability is not necessarily the same as indeterminism (understood here as the existence of ontological probabilities). Classical chaotic systems show us that, while the underlying laws can be perfectly deterministic, the actual behavior we observe can be highly unpredictable, due to the extreme sensitivity of the system to the initial conditions. Thus, every time we observe a physical system behaving in an unpredictable way (including a quantum one), we can always assume that there is a hidden deterministic law governing its behavior. Thus, from a merely logical standpoint, one could argue that it is a matter of metaphysical taste to decide between determinism and indeterminism. Which criterion should we use to decide?

We claim that *evidence* is of the essence here. What is important for us is that there is much evidence that unpredictability in classical systems can be diminished by refining the experimental conditions. Moreover, this is true even for chaotic systems: no matter how short it is, we need to wait a finite amount of time to observe a separation between two given trajectories. If the initial conditions are suitably refined, we will have to wait a long time. Empirical evidence supports the following claim: for the so-called classical systems, unpredictability can be mitigated by refining the experiments. In other words, there is no evidence of any physical law forbidding us to assume that unpredictability can be reduced, in principle, by improving experimental conditions. Moreover, classical unpredictability exhibits a variety of different regimes and probability distributions. There is no universal law that serves all classical systems at once.

In contrast, in quantum physics, the finer the experiments, the higher the probabilistic behavior will be. The peculiar features of quantum probabilities manifest themselves more clearly with the development of more sophisticated preparation and detection devices. Quantum technologies rely heavily on that progress. Contrary to classical physics, empirical evidence suggests that quantum probabilities are irreducible since there is no sign that the refinement of experimental conditions will be reflected in an attenuation of the probabilistic behavior. Differently from chaotic systems, quantum probabilities appear in the form of universal mathematical laws, which apply in very different scenarios. These laws can be very precisely formulated and empirically tested. If there is some hidden determinism, there is no scientific evidence of it (after around 100 years of hard efforts). The founding architects of quantum mechanics realized this very fast, and that was, perhaps, one of their main achievements. Instead of investing their time in looking for hidden variables and hidden trajectories, they just realized that something radically new was going on in quantum phenomena. They adopted the hypothesis that there is a fundamental randomness in nature and that we can have robust control of quantum systems by determining the laws that govern the observed probabilities. One could say that they heard the message of experimental evidence [37], and they acted accordingly, creating one of the more successful theories of human history.

### 2.3. How to Avoid Empirical Evidence and Return to an Ignorance Interpretation of Probabilities?

However, there are (and were) several relevant authors who did not like the idea that there is intrinsic randomness in nature—even if this assumption is not supported by evidence. Starting with Einstein, who claimed that “God does not play dice”. According to the legend, Bohr replied: “Einstein, stop telling God what to do!”. Einstein advocated for the completion of quantum theory using hidden variables. That quest led to many interesting questions and developments. Among them, one can find Bohmian mechanics. According to the standard formulation of this theory, one can think of quantum systems as localized particles that interact in a non-local way through a sort of pilot wave. Due to a very intricate mechanism, the interaction is such that non-localities are canceled in such a way that we cannot use quantum systems to send signals faster than the speed of light. Moreover, some authors assume that even if the underlying ontology of Bohmian mechanics is fully deterministic, there is no way to use this determinism to make predictions about experiments. This is also the case in other interpretations. Therefore, even if a hidden deterministic dynamics is assumed in many of them, the probabilistic characteristics of experiments seem to be common features of the different approaches.

There is more than one version of Bohmian mechanics. As there is no way to tell how many angels fit inside the head of a needle, science poses no limit or criterion for deciding between metaphysical alternatives that are impossible to observe and control by assumption (and for which no empirical evidence can be collected). Hidden variables seem to provide a purely formal solution to the philosophical problems of quantum mechanics, given that they have the peculiar feature of creating infinitely many new unsolvable problems. Of course, everything would be different if, out of the assumption of hidden variables, there were some evidence or non-trivial prediction that could be tested.

Another development related to the quest for hidden variables is that of Bell inequalities. Attempting to follow Einstein’s path, J. Bell wondered whether there could be a local realistic explanation of quantum physics. This question led him to the discovery of what we nowadays call *Bell-type inequalities*. Empirical evidence seems to suggest that these inequalities are violated and that there is no hope for the non-contextual hidden-variable theories assumed by Bell. A great part of the physics community received these results as follows: “Bohr was right. It is pointless to keep looking for hidden variables. Complementarity is of the essence for the goal of understanding quantum mechanics”. However, it also led several authors to conclude that nature is non-local. Again, the supposed non-locality is such that there is no way to use quantum systems to send signals faster than the velocity of light. It is there but, in a sense, it is hidden from us. The celebrated non-local character of quantum physics is far from conclusive since it is clearly possible to develop *contextual* hidden variable theories, which are perfectly local and realistic (see for example [38,39]). However, all of this research did not prevent some philosophers from extracting the conclusion that the world is non-local (we refer the reader to references [40,41,42,43] for an example of the heated debate around this point).

The standard version of quantum theory poses two types of time evolution, the unitary one (which applies when systems are ideally isolated), and the collapse, which assumes that uncontrollable and irreversible changes of a physical state take place each time a quantum system is situated in a concrete empirical context. It is in the latter case—during the passing from the potential to the actual—that the intrinsic randomness manifests itself. Sometimes, the discontinuous change of state occurs naturally (as in the decay of an atom and the act of photon emission). In other situations, it occurs because we do it on purpose, by setting some specific experimental conditions to gain knowledge about a specific system. The assumption of a unitary evolution is a convenient idealization, given that quantum systems are never really isolated, and they can only exist in concrete empirical contexts. This is checked every day when samples are taken from quantum computers, which are, perhaps, the most controlled quantum systems ever built. Notwithstanding, unitary evolution works as a very good approximation in many situations of interest (for example, ions in a vacuum chamber at very low temperatures). We will not go into the details, but it is important to call the attention of the reader to the current research regarding the assumption of unitarity [44,45]. Moreover, with regard to the dynamics, we should mention that the so-called *entropic dynamics approach* commits to the ontic variables while, at the same time, it denies the ontic dynamics for them. The dynamics of probabilities are purely epistemic [46].

In general, the goal of killing the idea that there exists intrinsic randomness in nature is connected to what can be called “unitary quantum mechanics”. If all evolutions are unitary at the fundamental level, then nature is ultimately deterministic. Therefore, quantum jumps and any other manifestations of probabilistic behavior will be epiphenomena of a deterministic dynamic evolution. Moreover, many authors disliked the idea that there could be two types of dynamic evolution. Thus, they engaged in a quest for a quantum theory without collapse. The goal of this research program is to explain the measurement process (and the fact that the immediate reality surrounding us is classical) using only unitary interactions between the system and its environment. However, it is methodologically important to distinguish what is established as a scientific theory, and the research programs that are still in progress trying to replace it (or reformulate it).

The idea of developing non-collapse versions of quantum theory is nice, but the path is quite difficult. The strongest and most widely spread version of unitary quantum mechanics is based on a detailed study of the interaction between the system and its environment. These interactions can explain the preferred bases during measurements. However, it is still hard to explain the probabilistic behavior and why one observes a discontinuous change of state during measurements. For an up-to-date exposition of the problems involved and the state of the art of the proposed solutions, see [47,48]. The very idea of describing a macroscopic system as a simple mereological sum of quantum microscopic parts interacting unitarily is rather ambitious. There is a clear reductionistic assumption underlying it, and a philosophical analysis clearly warns against taking this assumption as a naive one. See [49] for further debate on this point. Moreover, it is worth mentioning that several authors have considered the so-called *measurement problem* as a pseudo-problem [50] (see also [51]).

The rejection of collapse does not necessarily lead to unitary dynamics. For example, the idea of describing non-unitary dynamics without appealing to collapse was studied extensively by I. Prigogine and the Brussels–Austin school [52,53] (related to this, see also [54], for a more recent review on quantum unstable systems). However, in the philosophy of physics community, the irreversible approaches are usually not taken into account, favoring alternatives that appeal to unitary evolutions only. One could say that there is a widespread belief that physics, at a fundamental level, is (or should be) unitary. This is a rather unfortunate situation, given that the assumption of intrinsic irreversibility in nature offers an interesting interpretational alternative. As an example of this, it can be used to describe the Loschmidt echo [55]. Notwithstanding, the so-called dynamic collapse approaches have gained more attraction.

The quest for hidden variables and unitary interactions has also led to very radical ideas, such as the assumption that many worlds (parallel to ours) exist, and that a “branching” occurs each time we perform a measurement. According to some versions of this approach, quantum probabilities are only subjective (i.e., there is no intrinsic randomness in nature). We are in a particular world and there is a very different version of us in a very different world. However, there is no way we can have a chat with our twins. In this interpretation, even if the probabilities are subjective, it is not possible to predict the outcomes of the quantum experiments.

However, despite all of the heated debates about hidden variables and hidden worlds, the assumption that there is intrinsic randomness in nature is still very strong among physicists and engineers. So firm is their belief in that assumption, that they are willing to devise, sell, and buy quantum random number generators. Even among those who still believe that there is a hidden determinism, it is widely accepted that it cannot be accessed and controlled. It should come as no surprise that the mathematical laws that govern quantum probabilities have very peculiar features, which we will discuss in what follows.

It is not our aim here to make a full review of all the positions regarding the interpretation of quantum physics (and even less to choose among them). The goal of this work is to pose some philosophical questions regarding the above-mentioned belief in the true randomness associated with quantum systems, and to analyze the development of quantum technologies under the light of one of the main threats of quantum probability, namely, quantum contextuality.

## 3. The Main Features of Quantum Probability

In this section, we formulate (very briefly) the axioms of classical probability and the analogous axioms for quantum and quantum-like probabilities.

### 3.1. Kolmogorov’s Axioms

Consider an empirical domain in which different events might occur. Let us represent all these events by the set Ω. For example, in a die toss, we have Ω={1,2,3,4,5,6}. There are propositions associated with those events, such as “the result is even”, “the outcome is odd and strictly greater than 1”, and so on. Such propositions are naturally represented by the subsets {2,4,6} and {3,5}, respectively. One can form other propositions, and perform logical operations on them. Endowed with these operations, Ω becomes a *Boolean algebra*. Consider then the Boolean algebra Σ formed by all possible subsets of Ω. A probability measure should assign a number in the interval [0,1] to each element of Σ, so each proposition has a definite probability of occurrence. These intuitions are captured in what is known as *Kolmogorov’s axioms*. A probability measure μ will be a function satisfying:

(1) μ:Σ→[0,1]

such that:

(1) μ(Ω)=1
(2) For any denumerable family of pairwise disjoint sets ${\left\{A_{i}\right\}}_{i\in I}$
  $$\mu \left(\underset{i\in I}{⋃}A_{i}\right)=\sum_{i}\mu \left(A_{i}\right)$$

The triad (Ω,Σ,μ) is known as a *probability space*. The set Σ is a σ-algebra (but the important thing for us is that it is also Boolean algebra).

A random variable *f* can be defined as a function from Ω to the reals, such that the pre-image of any Borel set is measurable (according to μ):

(2) f:Ω→R

such that for every Borel set, Δ⊆R,

(3) $$f^{-1}\left(\Delta \right)\in \Sigma$$

Random variables can be used to model observables of classical physical theories. Let us consider again the die example. Call *f* the observable that yields 1 each time the outcome is even, and −1 each time the outcome is odd. Therefore, each value of the observable has a definite probability. If the die is not loaded, the probability of obtaining outcome 1 is $p_{f=1}=\mu \left(\left\{2,4,6\right\}\right)=\mu \left(\left\{2\right\}\right)+\mu \left(\left\{4\right\}\right)+\mu \left(\left\{6\right\}\right)=\frac{1}{6}+\frac{1}{6}+\frac{1}{6}=\frac{1}{2}$. The mean value of *f* is given by the formula $\langle f\rangle =\left(1\right)p_{f=1}+\left(-1\right)p_{f=-1}=\frac{1}{2}-\frac{1}{2}=0$.

A more sophisticated example is given by the probabilistic description of a classical one-dimensional point particle. Assume that its phase space Ω is formed by two real coordinates *P* and *Q* (thus, $\Omega =R^{2}$). In that case, one can assume that the probabilistic description is given by a density function ϱ with Lebesgue integral $\int_{R^{2}}\varrho d\mu =1$ (where μ is the Lebesgue measure), such that, for each observable $f:R^{2}⟶R$, one has $\langle f\rangle =\int_{R^{2}}f\varrho d\mu$. Here we assume that, for every Borel set Δ, $f^{-1}\left(\Delta \right)$ is Lebesgue measurable. If that is the case, each proposition of the form $f_{\Delta }\;\;=$ “the value of *f* lies in the interval Δ” will have a well-defined probability value, given by the formula $p_{f_{\Delta }}=\int_{f^{-1}\left(\Delta \right)}\varrho d\mu$. Quantum mechanical observable mean values and probabilities are computed using analogous formulas, which are a natural generalization of Kolmogorov’s axioms (see the comparison between classical and quantum observables discussed in [56]).

### 3.2. Quantum Contexts and Quantum States

Incompatible experiments exist in quantum mechanics. There cannot be sharp measurements of positions and momenta in the same experimental setup. Similarly, it is not possible to measure the spin of a spin $\frac{1}{2}$ system in two different directions. Notice that this implies that two incompatible experiments give place to different probability distributions and random variables. This strong dependence on the context of measurement can be called *Bohr contextuality* and is closely related to the notion of incompatibility of observables [57].

**Figure 1:**
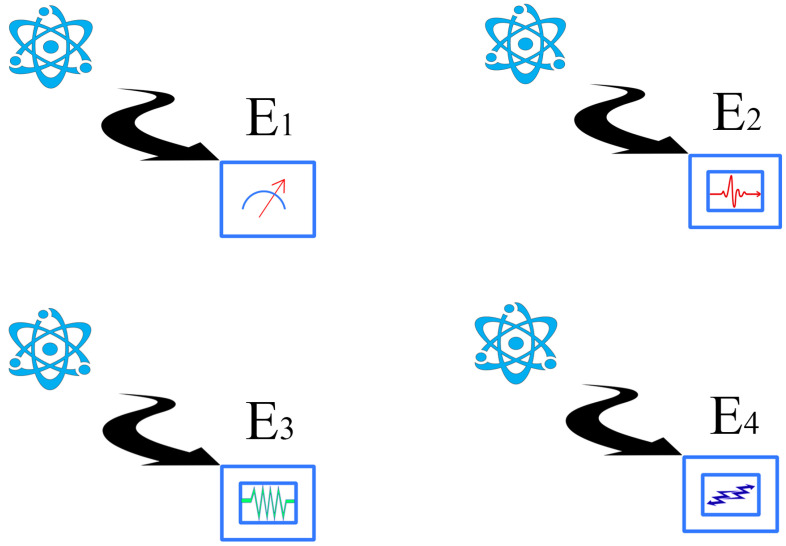
Four different experiments on a quantum system. Each one is assumed to have a different content from the others.

**Figure 2:**
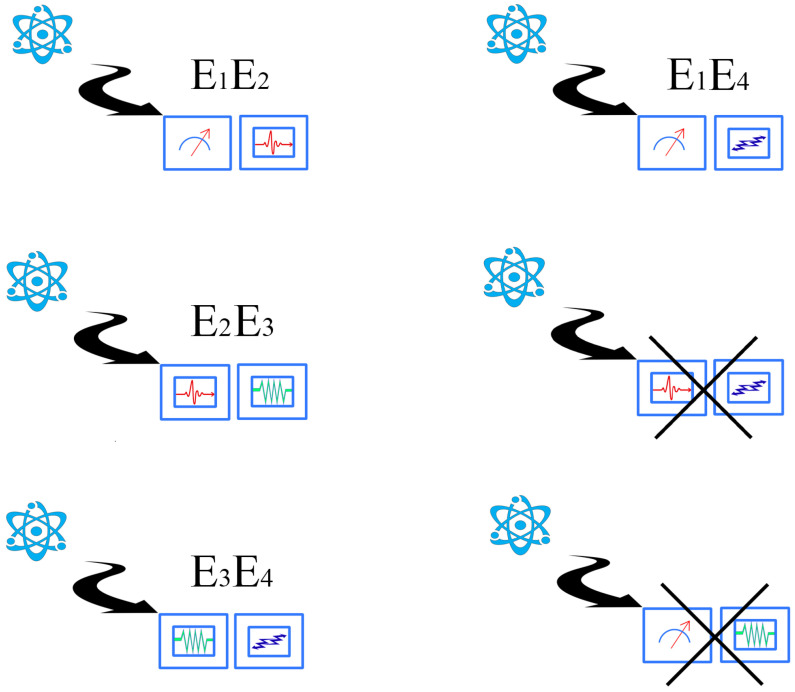
Some experiments can be jointly performed, meaning that the actions needed to implement them can be made at the same time in the same system. For other experiments, this cannot be done. This is an example of *incompatibility* between experiments.

**Figure 3:**
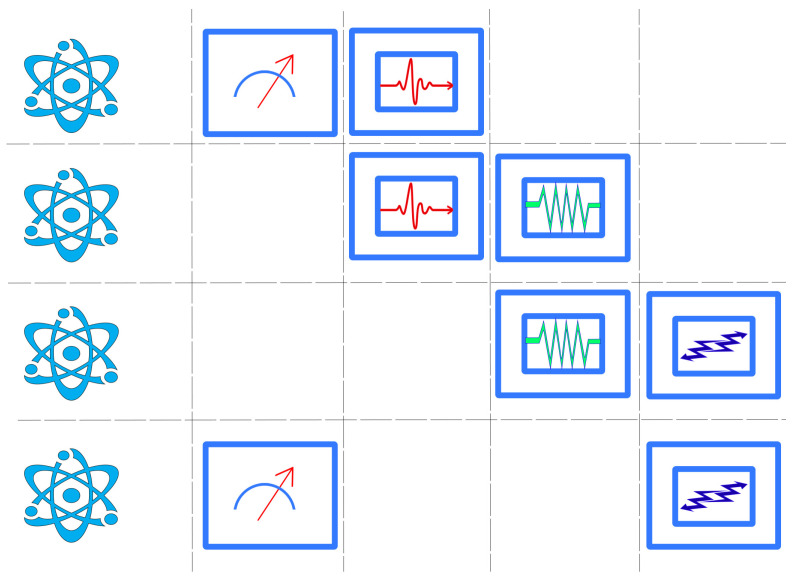
There is a joint probability distribution for the observables contained in every row (i.e., they are compatible). However, experiments taken from different rows cannot be jointly performed (due to the existence of incompatible observables). Furthermore, there is no global probability distribution for all possible experiments (this is an expression of contextual behavior).

Let us illustrate the situation in an intuitive way, by making some drawings. In Figure 1, we show a quantum system in which at least four non-equivalent experiments can be performed. Each of these experiments is assumed to have a different “content”: for example, $E_{1}$ could be the energy, $E_{2}$ the spin, momentum, or position, and so on (this is why we use different drawings to represent them). They could also represent different spin measurements. The exact details are not important here, only the idea that they represent physical magnitudes of interest. It might well occur that some of these experiments can be jointly performed, and others cannot. As an example, one cannot perform a joint experiment for the position and momentum. In a two-qubit system, the experiments represented by $\sigma_{z}⊗\sigma_{y}$ and $\sigma_{x}⊗\sigma_{z}$ cannot be performed together either. This situation is illustrated in Figure 2. The whole picture can be summarized in the table-like Figure 3. There is a joint probability distribution for the experiments associated with each row of Figure 3, but there is no global joint probability for the four experiments. Notice that this simple example illustrates the connection between Bohr contextuality (a strong dependence of the physics with regard to the measurement context) and the practical definition of contextuality as the non-existence of a joint probability distribution.

Strong dependence of the physics on experimental conditions, the impossibility of realizing joint experiments, and the non-existence of a joint (Kolmogorovian) probability distribution for all possible contexts are, in principle, three different notions that, in quantum physics, are deeply intermingled. We will see in brief that these notion can be given a very precise mathematical formulation. Notice also that we did something on purpose: the contexts were chosen in such a way that an experiment could appear in two different contexts. Such experiments have the same content but are jointly performed with other experiments that might be incompatible. This is a crucial feature of quantum mechanics: the empirical contexts—even if they can be incompatible—are intertwined by experiments that have the same content. Again, this fact will have a very definite mathematical expression below. Such a universal mathematical formulation has no analog in contextual probabilistic models taken from other research areas (different from quantum mechanics).

We have used a representation appealing to images, in order to highlight how the mathematical formalism emerges from the physical aspects of the problem. Let us now look at the mathematical description with more detail. For a given physical state of the system, which can be considered a result of a precise preparation procedure, we have definite probabilities for each experiment. Under ideal conditions, the theoretical probabilities, which are predicted by quantum theory, coincide with the observed frequencies associated with each outcome. Out of them, one can compute empirical mean values. According to quantum theory, the probability associated with an experimental result can be computed as

(4) $$p\left(P_{\Delta }^{A}\right)=tr\left(\rho P_{\Delta }^{A}\right)$$

where ρ is the density operator representing the physical state of the system and $P_{\Delta }^{A}$ is an orthogonal projection representing the empirically testable proposition “the value of the observable *A* lies in the interval Δ”. Here, *A* is a self-adjoint operator. This is the Born rule for computing probabilities in quantum theory. The mean value of the observable associated with *A* is given by

(5) 〈A〉=tr(ρA)

If there are not too many experimental imperfections, the observed frequencies and mean values tend to be the theoretical ones as the repetitions of the experiment tend to infinity (and this assumes the preparation of a large number of copies of the physical state). For each experiment, we have a classical (Kolmogorovian) probability associated, and each observable can be described as a classical random variable. Assuming that each context has two observables, let us call $f_{i,j}$ to the random variable corresponding to the *j*-th experiment when considered in context *i*. Associated with the diagrammatic description of Figure 3, there is a mathematical Table for the random variables (see Table 1).

As stressed above, some experiments are repeated in different contexts. We must be very careful here, given that a subtle problem appears. Should we identify the repeated experiments? Should we use the same random variable to describe them? In principle, they are different, given that nobody grants that the change of context allows one to preserve the identity of the results. Let us dig deeper into the phenomenology of the matter. Suppose that we perform experiment $E_{1}$ jointly with experiment $E_{2}$. That amounts to executing a concrete list of tasks, which are situated in a definite space-time interval. One example of execution could be the following: rational agent $A_{1}$ perform the tasks associated with $E_{1}$ and agent $A_{2}$ performs the tasks associated with $E_{2}$, and this is done in the space-time interval $\Delta_{1,2}$ (notice that the agents could be machines, given that, nowadays, experiments are, to a great extent, automated). If we now consider $E_{1}$ jointly performed with a different experiment $E_{4}$ (which is incompatible with $E_{2}$), the associated tasks should be necessarily carried out in a different space-time interval $\Delta_{1,4}$, and possibly, not necessarily by the same agents. Therefore, the tasks associated with experiment $E_{1}$ jointly performed with $E_{2}$, are—by force of the laws of quantum physics and the principle of complementarity—not exactly the same as those associated with $E_{1}$ jointly performed with $E_{4}$. If an option is chosen, the other must be necessarily counterfactual (or just a repetition in an equivalent system). Notice also that, rigorously speaking, the system might not be necessarily the same, because, at least, if it is not just an equivalent copy (as is usual in quantum experiments), it must be considered at a different time. Usually, a natural assumption is made when we consider a system at different times, namely, *that we are speaking of the same system*. However, this is a non-trivial hypothesis in view of the philosophical issues related to quantum indistinguishability [58]. For these reasons, it is reasonable to make the cautious move of indexing the random variables with labels that make explicit their context of appearance. The random variable $f_{1,1}$ is the one associated with experiment $E_{1}$ considered in context 1, while $f_{4,1}$ represents the random variable associated with the experiment $E_{1}$ when considered in context 4. In principle, their associated probabilities and mean values could be different, depending on the context in which they are considered. This is what actually happens in fields of research such as mathematical phycology [59] (see the related discussion posed in [60]). However, something really amazing happens in quantum physics. The theory predicts that the marginal probabilities associated with a given experiment do not depend on the context in which it is considered (but see the discussion of experiments presented in [57]). This peculiar feature is called a *no-signal condition* and can also be given a very precise mathematical formulation.

What are we going to do then with the repeated experiments? Should we identify them? This is a very subtle problem since, even if completely equivalent, they are by force *not the same*. In general, a natural identification is silently done in physics. Random variables with the same content (but belonging to different contexts) are made equal without further discussion. In a recent series of works [58,61,62], it was proposed that repeated experiments are neither equal nor different. They are considered *indistinguishable*, the latter being a notion taken from non-reflexive logic. The identity of random variables is a highly non-trivial matter, and this sort of “identification principle” is at work in quantum theory in a very specific way. As a result, quantum contexts are intertwined by the indistinguishability relations among repeated experiments [63].

The intertwining of contexts is important for two main reasons. The first one is that it gives place to a technical research field whose goal is to characterize the family of possible contexts of a quantum system as a definite algebraic structure. It can be described as a pasted family of Boolean algebras (that gives place to a non-distributive orthomodular lattice) [64]. The second implication is that the intertwining lies at the basis of a major result in the quantum foundations literature: the Kochen–Specker theorem [65,66]. Any truth value assignment for quantum propositions—satisfying a very natural functionality condition—gives place to a contradiction. Therefore, the Kochen–Specker theorem implies that it is not possible to reasonably assign context-independent truth values to quantum propositions (and this is known as the Kochen–Specker contextuality). This feature is also the basis for the derivation of inequalities that can be tested empirically. See, for example, [67] and the references therein for recent major results in this regard. For a recent review on the subject, see [68]. Quantum randomness was related to the contextual and discrete nature of quantum physics in [69] (see also [70]).

Summarizing, we have:

- One can perform different experiments on a quantum system (trivial).
- Some of these experiments can be jointly performed. Others cannot (this is a non-trivial quantum feature).
- There is a joint probability distribution (that obeys Kolmogorov’s axioms) for experiments that can be jointly performed. However, the physics changes radically from context to context (consider the wave-particle behavior in a Mach–Zehnder interferometer): the strong dependence on the context is an expression of what can be called Bohr contextuality [57].
- There is no joint (Kolmogorovian) probability distribution for all possible contexts. This is a mathematical expression of the notion of contextuality.
- Experiments with the same content appear as repeated in different (incompatible) contexts (for quantum models of dimensions greater or equal to 3). This happens with a high degree of regularity, and can be given a very precise mathematical description. This is a highly non-trivial feature of quantum physics, which has no classical analog; a sort of “identification principle” seems to be at work here. As a result, empirical contexts are “intertwined” in a complex way. The characterization of this intertwining is one of the main challenges for understanding quantum contextuality.
- The marginal probabilities associated with concrete experiments do not depend on the other experiments that can be jointly performed. This is a highly non-trivial feature of quantum theory and is known as the *no-signal condition*.

There have been many efforts to characterize the intertwining. For our purposes, one can consider L as a pasting of Boolean algebras, which gives place to a non-Boolean algebra.

Now, we analyze with more (mathematical) detail the facts that each experiment defines a random variable that has a Kolmogorovian probability associated with it; for some of these random variables, a joint probability exists. Summarizing Table 1:

- For each $f_{i,j}$ in Table 1, we have a probability space $\left(\Omega_{ij},\Sigma_{ij},\mu_{ij}\right)$.
- For each row *i* of the matrix, we have a joint probability space $\left(\Omega_{i},\Sigma_{i},\mu_{i}\right)$.
- $f_{1,1}$ and $f_{4,1}$ have the same content, but they should not be a priori identified (and a similar conclusion holds for the rest of the repeated random variables).

A concrete preparation of the system defines a physical state. Mathematically, a physical state will determine a Kolmogorovian probability distribution for each possible experiment (out of which we can compute mean values and correlations). Therefore, a quantum state can be defined as a collection of Kolmogorovian probabilities. Given all possible contexts conceivable, a quantum state defines a probability for each one of them. The situation can be described with the following diagram:

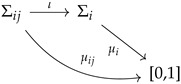

where $\Sigma_{ij}$ and $\Sigma_{i}$ are Boolean algebras associated with each experiment and each context, respectively. The injection arrow indicates that each experiment is contained in at least one context (physically: a maximally commuting set of observables; mathematically: a maximal Boolean algebra). Each $\Sigma_{ij}$ and $\Sigma_{i}$ has associated Kolmogorovian measures $\mu_{ij}$ and $\mu_{i}$ (and $\mu_{ij}$ is the marginal of $\mu_{i}$, computed in the usual way).

Using the above diagram, classical probability theory can be used to describe quantum systems in a quite natural way. What is the catch? Where are the so-called “quantum probabilities” we were promised (in the Introduction)? One could end up with the above diagram and describe everything using classical probabilities, which are familiar to the non-quantum physicist and, if one forgets about the philosophical questions, simpler to understand. However, this simple way of seeing things distracts us from the main features of quantum theory, given that there is so much more structure yet to be revealed. The situation is analogous to that of general relativity. One can imagine that the idea of a curved space-time description might be distasteful for some people. There is no need to worry because one can always give a flat space description of everything. After all, space-time looks flat if we look at it in small enough regions. Moreover, one can always describe the dynamics by adding corrective forces to the equations of motion. However, no working physicist would do this, for two reasons. The first one is that the mathematical descriptions of many relevant physical systems would become unnecessarily cumbersome. The second one is that one loses the insight that comes out of the revolutionary idea that space-time is curved (i.e., we lose a lot of valuable conceptual information). Something very similar happens in quantum mechanics. It is better to use the quantum probability calculus given by Hilbert space quantum mechanics (and the usual intuitions behind it) than returning to a classical probability formalism with the old classical notions. Even if not all quantum features are fully understood, the return to a classical ontology (and mathematical formalism) would be a drawback.

Thus, even if one can always make the trivial move of reducing everything to classical probabilities, it is crucial to realize that it is extremely useful to see things from a radically different perspective. The distinctive feature of quantum theory is that, mathematically, there exists a global object that condenses the information needed for all conceivable experimental contexts (this acquires the form of a universal physical law, namely, the Born rule). In quantum theory, the above diagram can be completed:

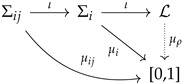



The global object is a non-distributive algebra L, which contains all possible contexts as maximal Boolean subalgebras. Very precise algebraic characterization can be given as the atomic and complete orthomodular lattice of orthogonal projections acting on a separable Hilbert space. For details on this algebraic notion, we refer the reader to [71] (see also [56] for a not-so-technical introduction to the subject). The empirical content of each P∈L should be very intuitive by now: the local Boolean algebras are formed by the mathematical representatives of the propositions associated with the different experiments one can perform on the quantum system, which are orthogonal projections. Contexts are nothing but maximal Boolean subalgebras (associated with complete sets of commuting observables). Over the global non-distributive algebra, there exists a measure μ, which, by appealing to Gleason’s theorem [72], can be proved to be equivalent to a density operator.

The above diagram can be reshaped in the form of axioms for a quantum probability calculus. A quantum state ρ can be given a description based in measure theory in terms of a function $\mu_{\rho }$ satisfying:

(6) $$\mu_{\rho }:L\to \left[0,1\right]$$

such that:

(1) $\mu_{\rho }\left(1\right)=1$
(2) For any denumerable family of pairwise orthogonal projections ${\left\{P_{i}\right\}}_{i\in I}$
  $$\mu_{\rho }\left(\underset{i\in I}{⋃}P_{i}\right)=\sum_{i}\mu_{\rho }\left(P_{i}\right)$$

where L is the orthomodular lattice formed by the orthogonal projection operators. Due to Gleason’s theorem, the above axioms define all possible quantum states (since there exists a bijective relation between density operators and such measures). In other words, Gleason’s theorem states that for every μ satisfying (6), there exists a density operator $\rho_{\mu }$ satisfying $\mu \left(P\right)=\mathrm{tr}\left(\rho_{\mu }P\right)$ for every orthogonal projection *P*. Moreover, vice versa, every density operator ρ defines one such a measure. The above equations constitute a natural extension of Kolmogorov’s axioms to the family of empirical contexts associated with a quantum system. This family can be structured as a non-Boolean algebra in which empirical contexts are represented as maximal Boolean subalgebras. They provide a measure-theoretic foundation for the quantum probability theory. The main difference between the quantum and classical cases is that Σ is distributive, while L is not. Thus, the quantum probabilities are *non-Kolmogorovian*.

Quantum mechanics is the first example of a non-Kolmogorovian probability calculus that has major relevance in the natural sciences and the technologies associated with quantum theory. Instead of using a Boolean algebra (or a sigma algebra), in quantum theory, we must use a non-distributive orthomodular lattice. It is remarkable that the problem of the axiomatization of quantum probabilities can be traced back to Hilbert’s sixth problem [56,73].

Before we continue, it is very important to mention different approaches that deal with the contextual behavior of quantum systems. For example, in the Vaxjo model for quantum probabilities [74], a family of classical probability distributions is assigned to a quantum system. Moreover, in the contextuality by default approach [60], the repeated experiments are not identified and are considered as different random variables (i.e., the intertwining is not realized, because there is no identification procedure or “principle” at work). These approaches provide different ways of describing general probabilistic models. However, these models can be described very naturally using measures over orthomodular lattices as follows. If we do not identify experiments with the same content but from different contexts, we obtain a family of Boolean algebras that have nothing in common. Without losing generality, one can identify the top and bottom elements of those algebras. This gives place to a global object that is an orthomodular lattice and is known as the *horizontal pasting of Boolean algebras*. Therefore, *every* family of Kolmogorovian measures can be seen as a measure over an orthomodular lattice. However, clearly, if one does not consider the intertwining (i.e., if no identification of repeated random variables is adopted), there is not much interest in creating a global object (from practical and philosophical considerations). The horizontal pasting can be used to describe signal models. The diagram associated with this perspective can be described as follows:

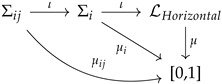

where $L_{Horizontal}$ is just the horizontal pasting of the local algebras (i.e., the possible intertwinings between contexts are not considered). We refer the reader to [56] for a review about generalized probabilistic theories in which the orthomodular lattices approach is explained in an accessible way.

Axioms (6) for quantum probability are not the end of the story: using the spectral theorem, we know that all possible self-adjoint operators can be written in terms of orthogonal projections. There is a ring of operators A associated with every quantum system, representing its physical observables. Thus, the final diagram reads:

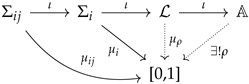



As we have seen, there exists no *classical* (Kolmogorovian) probability distribution for describing all possible experiments associated with a quantum system. However, there exists a global non-Kolmogorovian measure that is formally equivalent to the standard description of the quantum states using density operators. Thus, when physicists and quantum engineers perform their everyday calculations, they are putting the above-described non-Kolmogorovian probability calculus at work.

### 3.3. Negative Probabilities

There is another way to create a global object, which has major relevance for working physicists: negative probabilities (see [75,76,77,78,79] for examples of applications in quantum optics and quantum information theory). One can keep a global Boolean algebra, but the resulting global measure will fail to be positive. So, summarizing, if one aims to have a precise mathematical description of quantum states, there are at least two paths to follow:

- Alternative 1: paste the Boolean algebra and end up with a non-Boolean structure as we did in the previous section (and define the states as usual in the quantum logical approaches).
- Alternative 2: keep using Boolean algebras, but with negative probabilities.

In the negative probabilities approach, we obtain the following diagram:

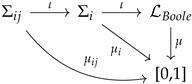

where $L_{Boole}$ is a suitably chosen Boolean algebra. For a recent account on this approach, which is based on measure theory, see [62] (but keep in mind that there are different ways of defining negative probabilities for quantum systems; see for example [75,80,81,82]).

Here, we briefly revisit the approach presented in [62] for the following reasons. It provides a general definition of *negative probability*, which is based on measure theory, extending Kolmogorov’s axioms in a natural way. Furthermore, it is independent of any Hilbert space structure. It also incorporates the notion of context from the very beginning and, from it, it is possible to inspire a contextuality measure that is easy to compute in practical applications.

In order to understand how the above diagram is completed, let us start first by giving the definition of a signed measure:

**Definition 1.**  *Let *Ω* be a sample space and *Σ* a σ-algebra over *Ω*. A* signed measure *is a function μ:Σ→R, such that*

(7) μ(∅)=0

*and for every denumerable and disjoint family ${\left\{A_{i}\right\}}_{i\in N}$*

(8) $$\mu \left(\underset{i}{⋃}A_{i}\right)=\sum_{i}\mu \left(A_{i}\right)$$

*The tripe (Ω,Σ,μ) is called a signed measure space.*

Out of that, we can define the analogous of a random variable for negative probabilities:

**Definition 2.**  *Let μ be a signed measure defined over (Ω,Σ), and let (M,M) be a Borel space with elements of M being real numbers, i.e., M is a σ-algebra over M. A (real-valued)* extended random variable *R is a measurable function R:Ω→M, i.e., for all Δ∈M, $R^{-1}\left(\Delta \right)\in \Sigma$.*

A signed measure μ defined over (Ω,Σ) is said to be normalized if μ(Ω)=1. Let $S_{\left(\Omega ,\Sigma \right)}$ be a collection of normalized signed measures defined over (Ω,Σ). We call such a set a *family of signed probability models*. A context will be a collection of extended random variables for which there exists a subalgebra in which the marginal probabilities are positive:

**Definition 3.**  *Consider a family of signed probability models $S_{\left(\Omega ,\Sigma \right)}$. Let $\left\{R_{i}\right\}$, i=1,⋯,n, be a collection of extended random variables defined on $S_{\left(\Omega ,\Sigma \right)}$. A* general context *is a subset $C_{j}={\left\{R_{k}\right\}}_{k\in N_{j}}$, $N_{j}\subset \left\{1,\ldots ,n\right\}$ of those extended random variables, for which there exists a sub-σ-algebra $\Sigma_{j}$ of* *Σ* *satisfying that, for all $\mu \in S_{\left(\Omega ,\Sigma \right)}$, by defining $p_{j}^{\mu }\left(F\right):=\mu \left(F\right)$ for all $F\in \Sigma_{j}$, the triad $\left(\Omega ,\Sigma_{j},p_{j}^{\mu }\right)$ becomes a probability space, and $R_{i_{k}}$ is a random variable with respect to it, for all $k\in \{1,...,n_{j}\}$.*

Once the notion of context is introduced, we can define a signed probability space, as one in which there exists a non-trivial context:

**Definition 4.**  *A* signed probability space, *also called a* negative probability space, *is a signed measure space (Ω,Σ,μ) endowed with a non-empty set of contexts $C=\{C_{j}^{\mu }\}$ (in the sense of the previous Definition), such that μ(Ω)=1. The measure μ in this space is a* signed probability *or* negative probability.

Most physical examples of interest are particular cases of the above definition (see [62] for more details). As in the previous section, random variables with the same content from different contexts are considered *indistinguishable*. Therefore, they are identified in the mathematical construction. Thus, we find again a sort of identification principle operating behind the construction of the global Boolean algebra. This is deeply connected with the fact that all the measures given in Definition 4 satisfy the non-signal condition. Out of the definition of negative probability space, it is possible to define a contextuality measure as follows. Given a family of contexts (defining a concrete contextuality scenario), one can find a signed probability which is compatible with them and minimizes the $L_{1}$ norm (for a given signed measure μ defined over an outcome set Ω, define ${∥\mu ∥}_{1}:=\sum_{\omega \in \Omega }\left|\mu \left(\omega \right)\right|$). Call $\mu^{⋆}$ to such a measure. Thus, the contextuality associated with the family of contexts is defined by the magnitude $\delta =(\sum_{\omega \in \Omega }|\mu^{⋆}\left(\omega \right)|)-1$ (see [62] for details).

The technical details of the properties of the negative probabilities and their associated contextuality measures reviewed in this section will be studied elsewhere.

## 4. Quantum Information Theory and Quantum Technologies: A Non-Kolmogorovian Perspective

It is important to start first with a definition of quantum information theory (QIT). A secure way to define it is as follows [83]:

**Definition 5.**  *The information theory that emerges from the assumption that the carriers of data are quantum systems.*

However, the above definition gives us a clue for providing a more precise technical definition. As we saw in the previous section, the transition from classical to quantum mechanics can be understood from the point of view of a change in the probabilistic framework. We must change Kolmogorovian by *quantum* probabilities. Moreover, as seen in Section 3, this transition implies the use of measures over non-distributive algebras. Therefore, one could define quantum information theory as:

**Definition 6.**  *The information theory emerges from the assumption that the probabilities are contextual and governed by axioms (6).*

Indeed, this possibility was discussed quite extensively in [11]. The latter definition already announces that quantum probabilities are at the core of the peculiarities (and advantages) of quantum technologies (see also [83] for a discussion on the role played by complementarity).

An important question is: can we simulate quantum probabilities (and therefore, quantum technologies) using classical systems? The answer might be somewhat surprising: yes, we can (see for example [10] and references therein). There is a trivial but important caveat: classical systems are not really quantum and, for that reason, they do not give place to a real quantum probability space. The subtle difference between the real and the imitation gives place to a big difference: efficiency.

All that we know today points in the direction that a classical simulator requires an exponential consumption of resources. In short, even if one can simulate a quantum computer with a classical one, evidence suggests that the cost of that move will be exponential (as the qubit numbers grow). Therefore, there is a huge difference between QIT and CI, and this difference also affects the efficiency of the associated technologies. We argue that this difference relies on the fact that the ontology of quantum mechanics is very different from the classical one. Or, in other words, from the point of view adopted in this work: quantum probabilities give place to a genuine quantum probability space, which entails genuine quantum contextuality. Recent results support this interpretation (see, for example, [84]).

Evidence to date suggests that the richness of the quantum state space and its dynamics cannot be reduced to a classical ontology. This has an expression that is crucial to understanding the whole idea of quantum computing and quantum supremacy: the belief in the falseness of extended the Church–Turing thesis [85]. We will come back to this point below after describing quantum computing in terms of quantum probabilities.

### 4.1. What Is a Computer?

Algorithms have been used for thousands of years. Euclid’s algorithm tells us how to compute the greatest common divisor of two integers. However, what is an algorithm? One could define it as an ordered set of operations that allows making calculations aimed at solving a specific problem. The good news is that any execution of an algorithm can be considered a physical process, and it is not necessary that any humans be involved in the tasks. One of the main signatures of our time is that we use physical systems (different from our minds) to implement sophisticated algorithms. Moreover, these devices happen to be programmable. We give them meaning by defining the relevant problems, introducing the inputs, and reading the outputs. This is the essence of nowadays multi-task computers. However, there is still something mysterious about them, given that they trace a link between logic and physics, and everything happens to work so well. Furthermore, since any information processing task has a physical substratum, it is expected that some features of it depend on the laws of physics (efficiency seems to be an example of this). In what follows, we will provide a schematic idea of what a computer is, to compare quantum and classical devices on a common formal ground. We will follow a variant of the approach presented in [12].

### 4.2. Deterministic Classical Computing

One could say that the main goal of a computer is to resolve problems. Usually, the problems that are of interest to us can be reduced to the task of computing functions of the form $F:{\left\{0,1\right\}}^{M}⟶{\left\{0,1\right\}}^{N}$. Such a function can be computed in terms of simpler functions, which are called *Boolean* (see, for example, [86], Chapter 2). The latter can be defined as maps that have strings of zeros and ones as inputs, and zero or one as outputs: $F:{\left\{0,1\right\}}^{M}⟶\left\{0,1\right\}$. A *Boolean circuit* can be described as a set of operations based on Boolean functions. If one chooses a suitable collection of Boolean functions, all other Boolean functions can be computed in terms of them. For example, one could choose the set A={∨,∧,¬}, where

**Definition 7.**  *$F_{\vee }:{\left\{0,1\right\}}^{2}⟶\left\{0,1\right\}$ with*

$$F_{\vee }\left(0,0\right)=0$$

$$F_{\vee }\left(0,1\right)=1$$

$$F_{\vee }\left(1,0\right)=1$$

$$F_{\vee }\left(1,1\right)=1$$

*$F_{\wedge }:{\left\{0,1\right\}}^{2}⟶\left\{0,1\right\}$ with*

$$F_{\wedge }\left(0,0\right)=0$$

$$F_{\wedge }\left(0,1\right)=0$$

$$F_{\wedge }\left(1,0\right)=0$$

$$F_{\wedge }\left(1,1\right)=1$$

*$F_{\neg }:\left\{0,1\right\}⟶\left\{0,1\right\}$ with*

$$F_{\neg }\left(0\right)=1$$

$$F_{\neg }\left(1\right)=0$$

Elementary Boolean functions can be instantiated by physical circuits. Moreover, these circuits are arranged in such a way that one can combine them and compute more complicated functions. This is how we can use a computer to compute a given function *F* (provided that we have enough memory space and CPU power).

A rational agent whose function is to manage the readout of the computation can only deal with (and communicate) truth values of the propositional structure defined by the power set $P({\left\{0,1\right\}}^{N})$. In this sense, the logic associated with a classical computer is represented by a Boolean algebra: the one generated by all possible bit strings in the set ${\left\{0,1\right\}}^{N}$.

### 4.3. Probabilistic Classical Computing

Given that quantum theory is essentially probabilistic, it should come as no surprise that quantum algorithms will be, in general, of a probabilistic nature too. Thus, in order to make a comparison between classical and quantum computers, it is important to review the idea of probabilistic classical computing [86]. Let us now provide a simple mathematical description of this model of computing, which uncovers the role played by Kolmogorovian probabilities in classical computing.

If probabilistic steps are allowed, to each input, $x\in {\left\{0,1\right\}}^{M}$, we do not necessarily have a deterministic outcome of the computation. Therefore, to each *x*, we must assign a probabilistic function $F_{x}:{\left\{0,1\right\}}^{N}⟶\left[0,1\right]$, satisfying

(9) $$\forall \;\;x\in {\left\{0,1\right\}}^{M}\;\;\;\sum_{y\in {\left\{0,1\right\}}^{N}}F_{x}\left(y\right)=1$$

The meaning of $F_{x}\left(y\right)$ is “the probability of obtaining the outcome $y\in {\left\{0,1\right\}}^{N}$ given the input $x\in {\left\{0,1\right\}}^{M}$ is $F_{x}\left(y\right)$”. Each $F_{x}$ defines a probability that obeys Kolmogorov’s axioms, with $\Sigma =P({\left\{0,1\right\}}^{N})$ (i.e., the Boolean algebra is given by the power set of the set of all possible strings of length *N*). Therefore, if a rational agent needs to handle the propositions associated with the readouts of our device, the underlying structure is Boolean. One of the main differences between classical and quantum computing is that the latter will rely heavily on its ability to exploit the non-Boolean structure.

### 4.4. Quantum Computing in a Schematic Way

Now we provide a schematic description of a circuit quantum computer with *n* qubits. In this model, states are represented by vectors $|\psi \rangle \in C^{n}$ (or, more generally, by density operators $\rho \in C^{n}\times C^{n}$), and the action of quantum gates is described by unitary operators $U\in C^{n}\times C^{n}$. With these mathematical tools at hand, the elementary sequence of a computation can be described by the following steps:

**Step 1**  We initialize the computer in the state $|\psi_{0}\rangle =|0\cdots 0\rangle$ (or any other conveniently chosen state).
**Step 2**  Next, we apply a collection of gates represented by unitary operators ${\left\{U_{i}\right\}}_{i=1,...,m}$, and obtain a final state $|\psi \rangle =U_{m}\cdots U_{2}U_{1}|\psi_{0}\rangle$.
**Step 3**  We perform measurements on a selected set of qubits. Depending on the results obtained, we decide which other steps to follow.

The above formalization works for noiseless devices (which do not exist in nature). A more realistic description of what happens inside a quantum processor must be then given as follows:

**Step 1’**  We initialize the computer in the state $\rho_{0}=\left|0\cdots 0\rangle \langle 0\cdots 0\right|$ (or any other conveniently chosen state).
**Step 1’**  Next, we apply a collection of gates. Real quantum gates are only approximately unitary. Thus, a final non-pure state ρ is obtained.
**Step 1’**  We perform measurements on a selected set of qubits. Depending on the result obtained, we decide which other steps to follow. Measurements (readouts) are also noisy (and this has to be considered too).

The concrete usage of a quantum computer involves, in principle, many of the above runs.

In many architectures, readouts are constrained to the computational basis $B_{0}{=\{|s\rangle \}}_{s\in {\left\{0,1\right\}}^{n}}$ (the *s*’s range over all possible strings of zeros and ones of length *n*). However, to obtain the full potency of quantum algorithms, the state evolution (given by the action of the unitary gates) cannot be restricted to elements of the computational basis. The relevant quantum algorithms generate superposition and entangled states that can be expressed as superpositions of the computational basis states. As it is a measurement basis, it should be clear that $B_{0}$ has associated a Boolean algebra of projection operators (see [12]).

Using the cyclic property of the trace, for every unitary matrix *U*, density operator ρ, and projection operator *P*, we have the equivalence:

(10) $$Tr\left(\rho U^{†}PU\right)=Tr\left(U\rho U^{†}P\right).$$

The above equation means that measuring in the computational basis is *mathematically* equivalent to a measurement in a different basis, provided that suitable unitary operations are applied. Notice that though there is a mathematical equivalence, the physical actions required are different. But the consequence of Equation (10) is that, when implementing quantum algorithms, we must use bases different than the computational one if some non-trivial quantum speed-up is hoped for. This point is very important and is related to the strength of a given quantum processor.

Universal quantum computing requires the capability of generating, in principle, all conceivable quantum algorithms. This is equivalent to having the capability of generating all possible unitary operators associated with the Hilbert space $C^{n}$ (for an *n*-qubits processor). A concrete device will be capable of instantiating only a subfamily of elementary unitary operators, which are known as *native gates*. For universal quantum computing, it is crucial that the elementary native gates form what is known as a *universal gate set*. Out of such a set, it is possible to simulate any other unitary operator. Noise will be the worst enemy of universality (but its effects can be mitigated with the use of error correction protocols and a high degree of gates and qubits control).

A universal gate set can give place, in principle, to any quantum circuit. Notice that this means that a quantum processor endowed with a universal gate set (+ error correction protocols), will be capable of approximating all possible states in $C^{n}$. This is equivalent to being able to navigate among all possible measurement contexts, covering the whole quantum probability space. Since any extant quantum computer will always have some degree of noise and imperfections, this goal can only be partially fulfilled. The strength of a quantum computer could, thus, be defined as how capable it is of generating all possible quantum states. The quantification of this capability is a non-trivial technical matter.

Each basis B of the Hilbert space, including the computational one, generates a Boolean algebra $P_{B}$ of projections that can be understood as propositions. Each algebra has associated a probability determined by the final state reached by the processor before a readout (a quantum state defines a non-Kolmogorovian measure over the orthomodular lattice containing all possible bases). The role of the orthomodular lattice of projection operators in some of the most important quantum algorithms is discussed in [87].

Let us now describe the main steps of a circuit quantum computer using an axiomatic framework that relies explicitly on the non-Kolmogorovian formulation of quantum probabilities given by Equation (6).

We start with an orthomodular lattice L, which represents the empirically testable propositions of our hardware. For the classical case, it is a Boolean algebra, but for the quantum case, it is the lattice of projection operators associated with the Hilbert space $C^{n}$ (if there are *n* qubits involved in the redout). The second element is a set of states C considered as measures assigning probabilities to each element of L. Again, in the classical case, these states obey axioms (1), while in the quantum case, they are given by all measures satisfying (6). The third element is a set *G* of automorphisms acting on L, representing the elementary (or native) gates of our quantum hardware (ideally, forming a universal set of unitary gates).

The set *G* is the generator of the collection P(G) of logical polynomials (by composition). We call the structure Ξ=〈L;C;G〉 a *generalized computational scheme*. The steps of an elementary act of computation can be described as follows (see also [12]):

- **Step 1**. Start with an initial reference state ν∈C.
- **Step 2**. Apply a collection of automorphisms ${\left\{U_{i}\right\}}_{i=1,...,m}$ to reach a desired final state $\mu \left(-\right)=\left(U_{m}\cdots U_{2}U_{1}\right)\left(\nu \right)\left(-\right)$.
- **Step 3**. Measure the system when state μ is reached, check the result obtained; depending on the result, stop the process or continue with the the other steps of the algorithm.

The above description of a circuit quantum computer suggests that quantum computing can be understood as a non-Kolmogorovian version of probabilistic classical computing. This perspective opens the door to investigating the role of contextuality in QC in a natural way. Indeed, in [87], it was stressed that the properties of the projective geometry associated with the Hilbert space was of the essence in quantum algorithms. All of this points to contextuality as the essential feature of quantum advantage, since, as we saw in Section 3, it is a physical phenomenon strongly related to the non-distributive character of the orthomodular lattice of quantum propositions. Our focus on the non-commutative probability calculus is in harmony with recent studies that suggest that contextuality is one of the essential resources allowing for the advantages of quantum computing [84,88,89,90,91].

## 5. The Extended Church–Turing Thesis and Quantum Supremacy

The *extended Church–Turing thesis* (ECTT), can be formulated as follows (see for example, [85]):

All computational problems that are efficiently solvable by realistic physical devices are efficiently solvable by a probabilistic Turing machine.

The ECTT can be interpreted as follows: every physical evolution can be efficiently modeled using a classical computer. R. P. Feynman was one of the first people who conjectured that there could be quantum systems for which time evolution could not be modeled efficiently using classical computers. Nowadays, we have good reasons to believe that this conjecture is true and that the ECTT is false. Quantum computers seem to outperform their classical cousins. Recent experiments support this idea [5,6,7,8,9]. Related to the failure of the ECTT, we must mention quantum supremacy. It can be defined as follows:

**Definition 8.**  *Quantum supremacy (or quantum advantage) is the goal of demonstrating that a programmable quantum device can solve a problem that no classical computer can solve efficiently.*

There is one important drawback in demonstrating quantum supremacy: the full-size realization of quantum computers remains challenging. Despite the spectacular advances in recent years, we are still far from the development of fault-tolerant universal quantum computers that are strong enough to solve problems, such as large integers factorization (Shor’s algorithm). In this context, several intermediate quantum computational models were proposed to show quantum supremacy and to give non-trivial information about the ECTT in the NISQ era. Some of these machines can be used to solve tasks that are believed to be hard for classical devices.

### 5.1. Quantum Random Circuits and Cross-Entropy Benchmarking

In 2019, Google announced that their Sycamore processor demonstrated quantum supremacy [5]. Sycamore had 53 qubits, which amounted to a dimension of the Hilbert space of $2^{53}$∼$10^{15}$. The idea of their experiment was to sample from a large quantum random circuit. This means that a family of circuits is generated randomly, by picking quantum gates from a universal set, and following a concrete pattern (see [5] for details). Each randomly generated circuit *C* is repeated and measured *N* times. As a result, a sequence of bitstrings ${\left\{x_{i}\right\}}_{i=1,...,N}$ is obtained. Each bitstring is a sequence of 0 and 1’s in length *n* ($x_{i}\in {\left\{0,1\right\}}^{n}$), with *n* being the number of qubits in the processor (n=53 in the Sycamore experiment). For each circuit, there are, in principle, $2^{n}$ possible bitstrings as outcomes. Given the circuit *C*, each $x_{i}$ has associated probability $w_{i}$, which is determined by the state generated by *C*. The probabilities associated with the sampled bitstrings are very hard to simulate classically. As a result, it is hard for a classical computer to simulate the sequence ${\left\{x_{i}\right\}}_{i=1,...,N}$, when *C* is randomly generated.

It is important to make some technical observations on quantum random circuits before continuing. First, an ideal task would be to set a quantum device to generate unitary operators taken from the Haar measure. Intuitively, this means that each possible random circuit is “drawn” from the set of unitary matrices with uniform probability (i.e., there is no bias for choosing any particular circuit). However, this task is exponentially hard even for quantum computers. Fortunately, it is possible to generate pseudo-random circuits, generating states that are “quantum enough” to obtain results that deviate from classical behavior. The generation of all possible unitaries following the Haar measure would imply that all possible quantum states are reached. By recalling what we saw in Section 4.4, this is equivalent to reaching all possible contexts in L. A real quantum computer will only be able to reach a subfamily of these contexts, but one that is big enough to realize a great deal of the non-Kolmogorovian state space. In light of what we have seen in Section 4.4, it should be clear that the quantum advantage studied in this experiment is directly related to the non-classical nature of quantum probabilities. The question is—how much do the probabilities depart from the classical ones?

It is possible to distinguish the sequences generated by a quantum random circuit from those generated purely at random (i.e., by using the uniform distribution). Relying on the *cross-entropy benchmarking*, it is possible to quantify the departure from the uniform distribution. The estimation performed in [5] yields that the time needed for a classical supercomputer to model the obtained sampling is 10,000 years.

The IBM team had another estimation; they claimed that it was possible to compute the same task in a much shorter time [92]. Further research revealed that their results could be improved [93]. Thus, the claims about “quantum advantage” should be taken carefully. Despite the heated debate and reasonable doubts, what is clear is that the 53 qubits of the Sycamore processor were capable of generating a quantum probability space that was big enough to give classical supercomputers hard work. In the words of the authors of [5]:

One may wonder to what extent algorithmic innovation can enhance classical simulations. Our assumption, based on insights from complexity theory, is that the cost of this algorithmic task is exponential in circuit size. Indeed, simulation methods have improved steadily over the past few years. We expect that lower simulation costs than reported here will eventually be achieved, but we also expect that they will be consistently outpaced by hardware improvements on larger quantum processors.

As the authors of [5] suggest, it is likely that, in the following years, we will see a race in which classical supercomputers will be at their limits due to ever-growing quantum processors. What is important for us is that the power of the Sycamore processor (and other similar devices) comes from its capability of generating random circuits (which are obtained from a universal gate set) with a high degree of coherence. Looking back to the discussion of Section 3 and Section 4.4, this could be considered equivalent to generating a rich enough set of quantum states. One could say that the strength of the Sycamore processor (and similar architectures) relies on its capability of generating (as much as possible) the quantum probability space.

Surely, the frontier that defines quantum supremacy can be considered diffuse since, given a quantum experiment, besides possible controversies regarding the estimation of the classical computational cost, one can always try to devise a stronger classical computer to make them even. However, it is clear that the classical computational costs of simulating the candidates for quantum advantage are high (and might become much higher in the near future).

Assuming that the ECTT is false, an important philosophical question emerges: what feature in the ontology of quantum theory is the reason for the quantum speed-up? First, it is important to notice that, even if this question originates and belongs to the plain field of quantum physics, it should be also of the utmost interest to the philosophers of physics. There seems to be something ontological related to the efficiency of quantum computers since it also seems that we can physically distinguish classical simulators by their exponential costs in resources. Therefore, among the questions raised by quantum theory, the explanation of the quantum speed-up (or the failure of the extended Church–Turing thesis) must be added to the list.

Let us conclude this section by mentioning another example that is of interest: Boson sampling. It was proposed as an alternative, using resources based on linear optics (this means that it is not impossible to realize them with current technologies). In 2021, Xanadu announced that their Borealis processor reached quantum advantage using Gaussian Boson sampling [9]. We will discuss the details of this example elsewhere. However, it is important to take into account that this model of quantum computer relies on quantum indistinguishability to fulfill its task. An approach focused on the connections between indistinguishability and contextuality was developed in a series of recent works [58,61,62].

### 5.2. Why Is There a Quantum Speed-Up?

Some years ago, it was not unusual to ask a physicist (or a philosopher of physics) about the reasons for the quantum speed-up, and obtain an answer that would go, more or less, as follows: “Quantum computers use superposition and entanglement to obtain a quantum advantage”. Or even bolder answers, such as “Nature computes the solution in all possible worlds, and then, one manages to extract a global answer to the problem of interest”. However, delving into the problem, one can find those answers doubtful. One possible reason for this doubt can be attributed to the following theorem (see [94] for details):

**Theorem 1.**  *A quantum circuit implementing only the following elementary operations can be simulated efficiently on a classical computer:*

- *Preparation of qubits in all possible computational basis states;*
- *All possible Clifford gates (Hadamard, controlled-NOT, and phase gate S); and*
- *Performing measurements in the computational basis.*

If the reader cannot grasp the technicalities behind the above theorem, let us rephrase it with more intuitive words. It affirms that one can build a quantum computer that generates entangled and superposed states and, still, its computing power can be efficiently simulated with a probabilistic classical computer. Thus, no quantum speed-up can be attained with such a circuit. Therefore, a possible conclusion could be: even if it is clear that superposition and entanglement seem relevant for quantum supremacy, those features are not enough (see [17] for a different point of view on this claim).

We explore the consequences of the above result for the interpretations of quantum theory. Some adherents of the many worlds interpretation, for example, could argue that the quantum computer performs the calculations of all possible inputs in parallel, using different worlds. However, we have seen that a quantum device could generate maximal entanglement and superpositions, and still, show no quantum advantage. Thus, the “many worlds parallel calculation” picture, while appealing, seems to give a quite erroneous point of view of the physics involved in a quantum computer. A similar remark can be made for Bohmian mechanics and its emphasis on non-locality.

Here, we argue that, on the contrary, it seems that it is the very nature of the quantum probability space that is responsible for the difference between classical and quantum computers. Of course, more research is needed to prove this thesis. Moreover, as we argued in Section 3, one of the main consequences of quantum systems obeying non-Kolmogorovian probability calculus is that quantum probabilities are contextual.

Indeed, several authors started to realize that, perhaps, the answer could be in contextuality [84,88,89,90,91,95]. As contextuality contains the violation of Bell-type scenarios as particular cases, one could imagine that this quantum feature might be the real reason behind quantum advantage. For example, in [84], it was proved that generalized contextuality is a necessary resource for quantum advantage. How does one interpret these advances? As we saw in Section 3, quantum contextuality can be considered a consequence of the fact that quantum systems obey a genuine quantum probability calculus. The author believes that this discussion suggests that it would be interesting to consider the thesis that, in the end, it is all about realizing a true source of non-Kolmogorovian probabilities.

Let us attempt to simplify the discussion of this section with bullet points by identifying the main ideas in question.

1. The power of quantum computers seems to rely on their capability of generating a large part of the richness of the quantum state space. Due to the Gottesman–Knill theorem, it is not enough to generate superposed and entangled states. Universal quantum computers need to generate a rich enough subset of the quantum state space.
2. Generating the quantum state space is equivalent to realizing all possible rotations implemented by unitary operators (quantum gates) in a coherent way. For this task, a universal set of gates is needed.
3. (Quantum) contextuality is directly related to the non-Kolmogorovian nature of the quantum probability calculus.

As we have shown in Section 3, the above points seem to be deeply connected. Many studies about quantum advantage consist in identifying which *resources* are needed for the success of quantum algorithms. Examples of such resources are entanglement, coherence, non-locality, and contextuality. However, the above discussion could indicate that the explanation might not be reducible to any of these alternatives on its own.

Keeping in mind the discussion presented in this work, it seems that the quantum advantage could be related to the ability of a quantum device to instantiate a rich enough family of non-compatible but intertwined measurement contexts. It is important to devise theoretical tools to numerically quantify this ability. In particular, we propose to interpret the cross-entropy benchmarking as an example of such a tool. It seems to be the global character of the quantum probability calculus what lies behind the speed-up of quantum computers. Thus, one might conjecture that the reason for quantum advantage can be stated as follows:

Quantum systems are the only sources of a true non-Kolmogorovian probability known to us. It is exponentially hard for a classical entity to efficiently simulate a sufficiently rich non-Kolmogorovian probability space. Therefore, quantum advantage can be defined as the ability of a quantum device in generating genuine quantum contextuality, which is directly related to its ability in exploring the quantum probability space by the application of a rich enough set of quantum gates.

Notice again that the above interpretational framework is very different from what one may extract from the ontologies associated with the Everettian or Bohmian reformulations of quantum mechanics. In those frameworks, probabilities can be interpreted in essentially classical ways (i.e., they are not ontological, given that both assume that nature is essentially deterministic). Here, we propose considering ontological probabilities and the peculiar laws they obey as the reason for the quantum speed-up. This interpretation gives a conceptual framework for understanding both recent theoretical results that point out contextuality as a sine qua non-resource for the quantum advantage and recent experiments that rely on sampling from quantum circuits.

## 6. Conclusions

In this work, we advanced the thesis that quantum systems are the only true sources of non-Kolmogorovian probability. The laws governing these probabilities are of a radically different nature to those of the classical ones, having deep implications for the debates on ontology and the technological applications of the theory. These assumptions might seem obvious to many, but not quite to others. Mainly, due to the existence of classical simulators and the fact that contextuality has been detected outside the domain of physics, for example, in cognition experiments. It is an open problem to establish up to which point the contextual behavior detected in cognition experiments can be compared with the quantum one. A cautious attitude is to consider them as different ontological natures (despite some mathematical similarities). Also, the technical details related to the quantum probability calculus, such as the Boolean algebras intertwining or the existence of an underlying non-Boolean structure, are unknown to many quantum information physicists and philosophers of quantum mechanics.

In this work, we presented arguments to conclude that:

- The probabilistic characteristics of quantum phenomena seem to have an ontological nature (this is the main working hypothesis underlying quantum theory). All empirical evidence to date supports this assumption, and it lies at the basis of the development of technologies that rely on genuine randomness.
- As such, the ontological probabilities involved in quantum phenomena are described by a highly non-classical probabilistic calculus. Quantum systems are, to our knowledge, the only examples of entities obeying those very precise mathematical laws. Therefore, quantum systems are the only genuine sources of a non-Kolmogorovian probability.
- Quantum information theory possibly emerges from the assumption that the devices used to store, process, and transmit information are entities obeying a genuine (not simulated) non-Kolmogorovian probability calculus.
- Quantum computing can be considered a non-Kolmogorovian version of classical probabilistic computing. From this perspective, the orthomodular lattice formed by the projection operators of the Hilbert space is an essential algebraic structure for understanding quantum advantage and quantum contextuality (see also [87]).
- The degree to which a quantum device is able to generate a non-classical probability space can be quantified by appealing to measures, such as the cross-entropy benchmarking (used in recent experiments attempting to demonstrate quantum advantage).

Obviously, more research must be devoted to determine the truthfulness (and usefulness) of the above assertions. In particular, to show (numerically and theoretically) how contextuality is involved in quantum advantage (which is the focus of ongoing efforts of diverse research groups around the world; see [84]). The author hopes to increase the attention of the reader on the relevance of addressing these questions, taking into account the non-Kolmogorovian features of quantum probabilities.

## Figures and Tables

**Table 1:** The different random variables associated with the experiments are organized in the table.

$f_{1,1}$	$f_{1,2}$	∅	∅
∅	$f_{2,2}$	$f_{2,3}$	∅
∅	∅	$f_{3,3}$	$f_{3,4}$
$f_{4,1}$	∅	∅	$f_{4,4}$

## Data Availability

Not applicable.
